# Reconfigurable Intelligent Surface (RIS)-Assisted Non-Terrestrial Network (NTN)-Based 6G Communications: A Contemporary Survey

**DOI:** 10.3390/s24216958

**Published:** 2024-10-30

**Authors:** Chika E. Worka, Faheem A. Khan, Qasim Zeeshan Ahmed, Pradorn Sureephong, Temitope Alade

**Affiliations:** 1 Department of Computing and Engineering, University of Huddersfield, Huddersfield HD1 3DH, UK; chika.worka@hud.ac.uk (C.E.W.); f.khan@hud.ac.uk (F.A.K.); q.ahmed@hud.ac.uk (Q.Z.A.); 2 College of Arts, Media and Technology, Chiang Mai University, Chiang Mai 50200, Thailand; 3 School of Computing Sciences, University of East Anglia, Norwich Research Park, Norwich NR4 7TJ, UK; t.alade@uea.ac.uk

**Keywords:** artificial intelligence, beamforming optimization, energy efficiency, high-altitude non-terrestrial platforms, machine learning, non-terrestrial networks, reconfigurable intelligent surfaces, 6G communications

## Abstract

This article examines the transformative potential of integrating reconfigurable intelligent surfaces (RISs) into sixth-generation (6G) wireless non-terrestrial networks (NTNs). The focus is on the RIS’s capability to address diverse user requirements, including secure data transmission, power efficiency, extended coverage, and enhanced data rates. The paper delves into the synergy between RISs and NTNs, emphasizing key components like multiple-input multiple-output (MIMO) systems and advanced radio communications. Additionally, it highlights the crucial role of artificial intelligence (AI) and machine learning (ML) in optimizing RIS-based beamforming to solve scientific and engineering challenges while ensuring energy efficiency and sustainability in NTN operations. By positioning RISs as a key enabler in shaping the future of wireless communication systems, this research underscores their significance in unlocking the full potential of NTNs and advancing next-generation wireless communications. This paper contributes valuable insights and projections for future research directions, highlighting RISs’ potential to revolutionize NTNs for 6G technologies.

## 1. Introduction

High-altitude platforms (HAPs), such as satellites and unmanned aerial vehicles (UAVs), are critical components of non-terrestrial networks (NTNs). HAPs operate at altitudes exceeding 20 km, surpassing traditional terrestrial networks (TNs), and possess the unique ability to transcend geographical barriers. As essential elements of NTNs, HAPs drive the advancement of wireless communication beyond terrestrial constraints, promising comprehensive coverage, enhanced connectivity, and adaptable functionality [1,2,3,4]. They are crucial in bridging the communication gap between remote or challenging terrains and enable diverse applications, ranging from aerial base stations (BSs) to cellular-connected NTNs [5,6,7,8,9].

In wireless communications, HAPs offer a strategic advantage due to their superior signal propagation capabilities and expanded coverage. This unique spatial positioning, combined with the flexibility of UAVs, makes HAPs dynamic and adaptive assets in the wireless communication arsenal [10]. Their deployment enriches NTN architectures, meeting the growing demand for connectivity in rural, oceanic, and challenging environments where terrestrial stations struggle to perform optimally [11]. This transformative impact of HAPs helps to address key challenges faced by terrestrial BSs. In scenarios with inadequate or compromised terrestrial infrastructure, HAPs offer agile and efficient solutions, ensuring uninterrupted connectivity. Their mobility and adaptability are particularly beneficial in emergencies, crowded events, or remote areas [12]. By serving as aerial base stations (BSs), HAPs redefine the concept of on-demand assistance to cellular networks, overcoming limitations associated with traditional TNs.

The dynamic and unpredictable nature of non-terrestrial BSs underscores the need for advanced coverage optimization solutions, highlighting the critical role of HAPs in the NTN landscape [13]. Machine learning (ML) algorithms are used in contrast to traditional mathematical optimization methods. This approach has become essential for efficiently managing resources and mobility in HAP stations. HAPs, acting as intelligent agents, leverage ML techniques within Markov Decision Processes to optimize trajectories of aerial platforms, deliver specific services, and meet demanding performance standards. This shift towards ML-driven control strategies reflects the evolving complexity of NTN communications, encompassing a broader range of non-terrestrial platforms beyond UAVs. These advancements underscore the significance of integrating ML into NTN architectures to enhance efficiency and adaptability in non-terrestrial communication systems [14,15,16].

**Figure 1:**
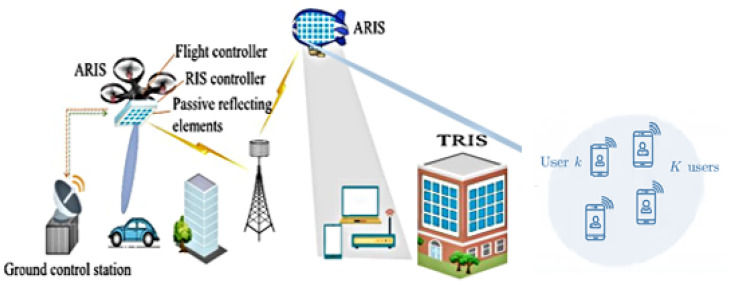
High-altitude platform (HAP) system architecture integrating ARIS for enhanced wireless communication for multiple users [13].

Reconfigurable intelligent surfaces (RISs) have recently emerged as a key enabling technology for 6G networks due to their capability to optimize wireless propagation environment, offering extended network coverage in a cost-effective manner. Integrating RISs into HAPs offers significant advantages, particularly in enhancing connectivity. Figure 1 depicts the scenario involving connectivity, where RISs control the phase of the incident signal, using one radio frequency (RF) chain to enhance the signal-to-noise ratio (SNR). As crucial relays between deep space networks (DSNs) and TNs, RISs facilitate efficient communication for interplanetary and Earth-orbiting missions, improving overall communication reliability and efficiency [17,18]. RIS-assisted HAPs provide significant advantages in coverage, connectivity, and energy efficiency. The dense deployment of HAP systems extensively enhances ground coverage, effectively eliminating coverage gaps and ensuring continuous connectivity, especially during handoff processes involving low-Earth-orbit (LEO) satellites [19].

This paper provides a comprehensive survey of RIS-assisted NTN-based 6G communications. The main novelty of our work is highlighted through addressing the following key questions:

1. What is RIS technology and how does it integrate with HAP systems?
2. Can conventional channel estimation methods and ML-based solutions be applied to RIS and NTN systems?
3. How can RISs and NTNs be integrated into 6G technologies?
4. How can energy efficiency and sustainability principles be applied to RISs and NTNs?
5. What are the future perspectives and challenges faced by RIS and NTN technologies?

**Figure 2:**
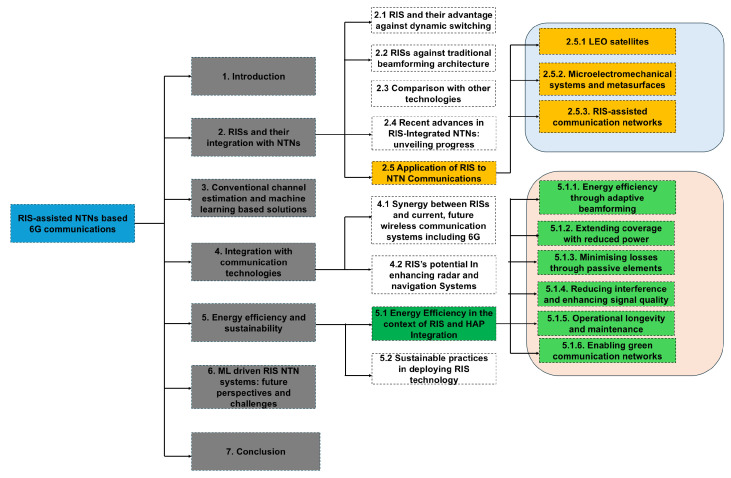
Taxonomy of RIS-assisted NTN-based 6G communications along with blocks illustrating the interconnection of research themes, forming a cohesive framework to accomplish RIS-assisted NTN-based 6G communications.

The details of the different topics covered in this paper are shown in Figure 2. The remainder of the paper is organized as follows. Section 2 discusses the basic principles of RISs, beamforming optimization techniques for RIS-HAP systems, and applications of RISs in NTNs. In Section 3, ML-based channel estimation solutions in RIS-HAP systems are elaborated. RIS integration with other communication technologies is presented in Section 4. Section 5 focuses on energy efficiency and sustainability issues in RIS-HAP systems. Section 6 explores the intersection of ML and RISs in wireless communication networks and discusses future perspectives and challenges in implementing RIS-based NTN systems. Finally, Section 7 concludes with a summary of the key findings.

## 2. RISs and Their Integration with NTNs

This section navigates the interplay between RISs and HAPs by understanding the core principles that govern the abilities of RISs to manipulate electromagnetic (EM) waves to enhance the performance of communication systems. We first define RISs and compare their superiority to dynamic switching. We then help to differentiate between RISs with traditional beamforming followed by its comparison with other relevant technologies such as relays, massive multiple-input multiple-output (MIMO) systems, etc. Finally, we highlight the advantages of integrating RISs with NTNs and their applications.

### 2.1. RISs and Their Advantage Against Dynamic Switching

RIS refers to a flat surface comprising several metamaterial reflection components with adjustable phase transitions, enabling manipulation of incoming EM waves’ phase shifts. This cutting-edge technology allows for the dynamic control of the wireless environment and identification of reflected EM wave behaviors [20]. Controlling transmitted signals via a reflection array was first introduced in 1997, creating reflection displays using metamaterial elements to manage millimeter wave (mmWave) reflecting surfaces effectively [21,22]. Their work suggests improvements in production, component design, and input layout while demonstrating the beamforming capabilities of reflecting panels in the millimeter wave (mmWave) range. Utilizing RISs can improve communication effectiveness significantly.

Various strategies have been employed in recent years to achieve metamaterial tunability, including embedding switching apparatus externally and integrating positive intrinsic-negative (PIN) diodes with metasurface components [23]. Nowadays, reconfiguration capabilities on metasurfaces have become achievable, and several research teams have developed prototypes. Studies have explored software-controlled passive patch antennas for static beam development, showcasing advancements in beamforming capabilities and reconfiguration in wireless systems [24,25]. Several advancements in RIS technology have been achieved recently, showcasing diverse capabilities and advantages:

1. A PIN-based 256-unit RIS with excellent energy density and cost-effectiveness has been developed [25,26,27].
2. Researchers have successfully regulated orientation and transmission reflection using a variable anisotropic computerized sequence alignment metasurface [28]. It was shown that doubling the number of antenna elements from 400 to 800 increased the achievable rate to 2 bps/Hz.
3. NTT Docomo from Japan has created a highly transparent two-dimensional metamaterial, allowing for covert usage in exhibitions and advertisements [29].

These advancements highlight the superiority of RIS technology over dynamic switching methods, as RISs offer several advantages, such as low power consumption and simplified interference management. This capability positions RISs as critical enablers for intelligent wireless environments, particularly for 6G [30,31].

### 2.2. RISs Against Traditional Beamforming Architecture

Compared to traditional beamforming architectures, a significant innovation of this scheme lies in its ability to fully harness the potential of RISs with their unique programmable feature as an external and standalone analog beamformer [32,33,34]. This eliminates the need for internal analog beamforming at BSs, leading to simplified architecture and a substantial cost reduction. The research discussed in [35,36,37,38,39,40], addresses the joint beamforming optimization for RIS-aided full-duplex (FD) NTN communication systems. Optimizing beamforming in RIS-aided FD communication systems is essential for enhancing performance across various metrics as it improves signal quality, spectral efficiency, and interference mitigation. In this optimization approach, refining beamforming vectors at both the transmitter and receiver ends and adjusting the phase shifts on RIS elements are jointly carried out. The most important objectives are to maximize the received signal power, minimize interference, and adhere to the communication system constraints, including the handling self-interference inherent in FD setups that share the same frequency spectrum [41,42,43]. To address these challenges comprehensively, diverse optimization algorithms and techniques such as convex optimization, genetic algorithms, particle swarm optimization, and deep learning (DL) methodologies are deployed. The benefits of RISs are fully harnessed to achieve superior spectral efficiency and interference management by synergizing the optimization of beamforming vectors and phase shifts in RIS-FD systems [44,45]. In [45], the presented framework showed a power reduction of 6 dB at 5 bps/Hz compared to the baseline method. Designing efficient beamforming algorithms considering self-interference and the needs of joint optimization further enhances RIS-aided FD system performance [39,40,46].

The study in [43] focuses explicitly on minimizing total transmit power by combining optimizing active beamforming at the sources and passive beamforming at an RIS positioned between them. The authors showed the effectiveness of RISs for FD schemes, showing that they outperform half duplex when the transmit power is less than 25 dBm. The number of transmit antennas was fixed to 40 and the data rate was set to 4 bps/Hz [43]. They proposed an efficient algorithm such as alternating optimization to solve the non-convex problem. In addition, joint optimization of beamforming, RIS phase shift, and user association in a multi-cell network aided by multiple RISs were studied [38]. The authors proposed less complex algorithms compared to existing methods and effectively exploited multiple RISs to improve network performance.

The process of joint beamforming optimization for RIS-aided FD communication includes simultaneously optimizing the beamforming vectors at the transmitter and receiver and adjusting the phase shifts of the RIS [47]. It was shown in [47] that the target sum rates of 9.5 and 12.5 bps/Hz can be achieved by saving 58% and 39% of the RIS elements, respectively. This optimization strategy aims to strengthen signal quality at the receiver, boost spectral efficiency, and mitigate interference in RIS-FD systems [48]. By jointly optimizing these parameters, the system can benefit from the RIS’s unique advantages, such as enhancing signal strength and directing signals toward intended users or nullifying interference.

### 2.3. Comparison with Other Technologies

The research in [49] explores an array of different solutions, uncovering significant SNR improvements in massive MIMO and RIS systems. Under far-field conditions, SNR grows linearly with the number of antennas (*N*) in massive MIMO but quadratically ($N^{2}$) with RISs. However, near-field complexities limit RIS’s asymptotic SNR despite its faster growth. It emphasizes RIS’s potential for enhanced SNR, scalability, and cost-effectiveness compared to massive MIMO. Table 1 gives a comparative overview of these technologies. RIS, massive MIMO, relays, and backscatter operate across several parameters such as duplex capability, the number of transmit RF chains, hardware cost, and energy consumption. RISs, operating through passive or active reflection, stand out with their FD capabilities and zero RF chain requirement, leading to low hardware costs and minimal energy consumption. In contrast, although offering full or half duplex options, massive MIMO and relay systems necessitate numerous RF chains, resulting in significantly higher hardware costs and energy usage. Backscatter, similar to the RIS in terms of low cost and energy needs, operates passively but generally underperforms compared to RIS.

### 2.4. Recent Advances in RIS-Integrated NTNs: Unveiling Progress

Recent advances in RIS-integrated NTNs highlight resource optimization and performance analysis developments. Researchers are delving into strategies for power consumption minimization, energy efficiency enhancements, and sum-rate maximization, utilizing optimization techniques. These studies span from exploring joint beamforming strategies to designing optimal phase shifts, showcasing the adaptability and efficiency of RISs across various scenarios [50]. Researchers actively explore secure cooperative communications and employ alternating optimization strategies in RIS-integrated NTNs. These multifaceted contributions extend to comprehensive performance analysis, with studies deriving expressions for outage probability, symbol error rate, ergodic capacity, secrecy, and connection probability [51,52]. This collective body of work illuminates the versatility and robustness of RISs in enhancing the efficiency, security, and overall performance of NTN landscapes [35,53,54]. The simulation results in [7] revealed that our proposed robust scheme significantly outperforms the RIS with random phase, the non-robust scheme, and the secure transmission scheme without the RIS, achieving improvements of approximately 8%, 17%, and 41%, respectively, in terms of friendly interference performance. From addressing fundamental link challenges to enhancing security and performance, RIS stands as a cornerstone in shaping the future of NTNs.

We critically examined beamforming methods customized for HAP systems equipped with RIS technology. Some of the core advantages found are as follows:

- **Enhanced Network Coverage:** In scenarios where direct line-of-sight (LoS) links are blocked or absent, RISs can create virtual LoS links between ground users and HAPs in air–ground networks, significantly improving communication quality and extending network coverage [8,55,56,57].
- **Dynamic and Smart Control:** Mounting the RISs on a HAP allows for the dynamic control of the RIS’s location, leveraging the HAP’s mobility. This mobility introduces a new degree of freedom in the RIS’s design compared to terrestrial RISs (TRISs) installed at fixed locations as shown in Figure 1 [58,59].
- **Expanded Wireless Coverage:** When installed on an HAP, the RIS will likely establish an LoS path for transmitting and receiving components. Unlike TRISs, which support communication between two nodes in half the space, an RIS on an HAP can expand wireless coverage and achieve full-angle reflection [60].
- **Ultra-wide Bandwidth in THz or mmWave Band:** An HAP and RIS can operate in the terahertz (THz) or mmWave band, offering an ultra-wide bandwidth to meet high-data-rate requirements [61]. The mobility of HAPs and reconfigurability of RISs efficiently compensate for the high path loss and blockages typical in THz or mmWave links [60].
- **Enhancing Communication Links:** An imperative motive for incorporating RISs into NTNs is their ability to surmount the intricate challenge of closing communication links. Designed explicitly for scenarios involving mobile ground users with low-directional antennas, RISs strategically compensate for path losses. This intervention significantly enhances signal reception, resulting in elevated data rates and an overall boost in system performance [59].
- **Physical-Layer Security and Reliability:** HAP communication often suffers from LoS blockage and vulnerability to eavesdropping. An RIS can mitigate external interference, enhancing physical-layer security and reliability. For instance, passive beamforming through an RIS directs beams away from eavesdroppers while strengthening signals for intended users. This approach improves security and reliability in HAP communication, primarily when airborne RISs (ARISs) support performance enhancement after optimizing HAP trajectory or location [13,59,62,63].
- **Enhanced Data Rate and SNR:** RIS-assisted HAP communication aims to boost the data rate and SNR. Factors such as the number of RIS-reflecting elements, RIS location, and HAP-hovering position are considered. Optimization approaches are adopted to enhance the received signal power at the HAP [16,60,64].
- **Mitigating Interference and Spectrum Reuse:** Coexisting with TNs brings forth the challenge of co-channel interference in NTNs. An RIS emerges as a strategic tool for interference mitigation, creating nulls or constructive interference in specific directions [65,66]. This proactive interference management minimizes disruptions, ensuring efficient spectrum reuse and safeguarding the integrity of both TNs and NTNs.
- **Energy Efficiency and Resilience:** Energy efficiency takes center stage in NTNs, impacting mission duration, costs, and sustainability. Operating passively without active transmissions, RISs become a key player in enhancing energy efficiency and fostering green communications [65,66]. Moreover, RISs contribute to NTN resilience by providing an additional layer of redundancy. RISs adeptly redirect signals in the face of obstacles, atmospheric conditions, or disruptions to terrestrial networks, ensuring continuous and reliable communications.
  This comprehensive examination illuminates the multifaceted contributions of RISs in shaping the future of non-terrestrial networks and their pivotal role in next-generation wireless communication systems [67,68].

### 2.5. Application of RISs to NTN Communications

Incorporating RISs into NTNs and upcoming 6G technologies presents distinct advantages. RIS’s ability to manipulate EM waves without conventional transceivers aligns well with the objectives of NTNs, particularly in reducing payload weight and power consumption, crucial factors in satellite operations [69]. For 6G, RISs can be pivotal in realizing ultra-reliable, low-latency communication and enhanced connectivity. Its energy-efficient nature and reduced hardware requirements offer an economical and sustainable solution for the expansive infrastructure envisioned in 6G. The unique properties of RISs, including improved signal propagation and coverage, address the challenges in NTNs and 6G, such as large coverage areas and signal attenuation, making it an innovative and viable technology for 6G networks.

#### 2.5.1. LEO Satellites

**Figure 3:**
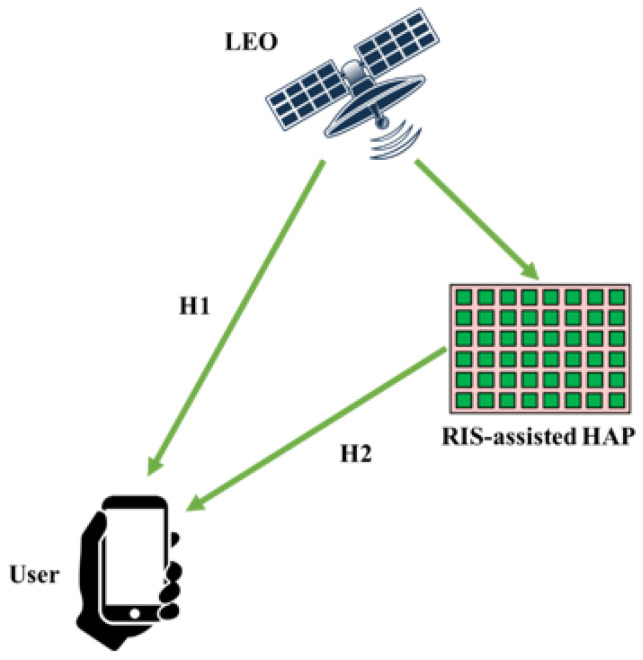
RIS-assisted HAP system model facilitating communication between a LEO satellite and a user via dual signal paths.

In 6G, LEO satellites may provide connectivity in remote and underserved areas. The LEO satellite has drawn the most attention these days among the various kinds of satellites working at varied altitudes in orbit because of its low height as well as the benefits that come with it, including less signal attenuation, a shortened transmission time, and a reduced expense in comparison to other satellites on a higher altitude [30,70,71]. The system model of an RIS-assisted HAP is illustrated in Figure 3, which includes an energy-constrained HAP with *M* antennas, an RIS equipped with *N* reflecting elements, and *K* single-antenna users [72]. The LEO satellite’s transmission bandwidth is still significantly greater than ground transmission, resulting in orders-of-magnitude of more significant route degradation. As a result, high-power broadcasters and high-sensitive receptors are typically used in LEO satellite technology to make up for the high route loss [73]. Nevertheless, doing so inevitably raises the expense and energy usage of the gear. As a substitute, the conventional passive reflect array has been used in satellite/deep-space telecommunications to accomplish unidirectional broadcasting for route loss mitigation without appreciably raising the equipment cost and energy usage [73,74,75,76,77].

#### 2.5.2. Microelectromechanical Systems and Metasurfaces

RISs or their various counterparts are the result of recent advancements in technology in microelectromechanical systems (MEMSs) and metasurfaces. These technologies have made it possible to repurpose passive reflections in real-time, significantly adjusting the inert transmission and reflection [78]. This development enables the creation of efficient, cost-effective wireless communication streams. Generally, RIS is a large EM metasurface composed of numerous, inexpensive static reflecting components. Each element can independently control the phase shift and frequency of incident signal, significantly boosting the power of the reflected signal in a desired direction, enabling fine-grained passive beamforming gains [79]. RISs are capable of handling the time-varying wireless channels of high-mobility LEO satellites and of compensating for the significant path loss caused by vast transmission distances thanks to its real-time tunability [80,81]. Moreover, unlike traditional active beamforming systems for satellite technology, RISs offer full-duplex, unobtrusive beamforming at minimal cost and power consumption, without the need for active transmitters or RF networks for signal processing or amplification [82].

#### 2.5.3. RIS-Assisted Communication Networks

Numerous studies on using RISs in different communication networks, including non-orthogonal multiple access (NOMA) and orthogonal frequency-division multiplexing (OFDM) systems, have been inspired by the RIS’s attractive characteristics [83,84,85,86,87,88,89]. The fundamental idea of RISs, where the RIS is installed on the satellite or close to the terrestrial users to improve their network throughput, has just been applied to satellite relay in some earlier research [90,91]. These works, nevertheless, assumed the ideal channel estimation for the design and operation of detached beamforming and neglected to take into account the crucial channel approximation/beam traceability problem for RISs, which remains a difficult task in practice owing to its numerous reflecting components that do not have data communication or processing capabilities, particularly for the scenario of LEO satellites with excellent mobility [92,93,94]. Even though effective passive beamforming models and channel coding strategies have been developed for various RIS-supported structures, most of these are focused on terrestrial communication networks with BSs and RISs fixed in position to serve steady-state users, which might not be suitable for high-mobility LEO satellite systems [95]. For example, the fast mobility of LEO satellites might lead to highly time-varying wireless channels with ground nodes (GNs) and satellites which can complicate regular channel estimation and beam management for both active and inactive beam positioning. Such challenges could significantly degrade communication performance [95,96]. Furthermore, the route identification and beam monitoring in RIS-supported LEO satellite communications are more difficult owing to the substantial Doppler impact generated by the LEO satellite’s rapid motion [96,97].

## 3. Conventional Channel Estimation and Machine Learning-Based Solutions

Classical optimization algorithms face significant challenges in meeting the demands of emerging applications in RIS-assisted integrated satellite (RIS-IS) operations and NTNs. These challenges primarily arise from the complexity of communication scenarios, unknown channel models, and difficulties in accurately modeling dynamic environments through mathematical expressions, which is further exacerbated by non-linearities due to hardware losses. The continuously changing configuration of RISs further complicates matters, hindering traditional methods’ applicability for real-time operations such as Internet of Things (IoT) or Internet of Vehicles (IoV) environments.

However, deep reinforcement learning (DRL), a combination of DL and reinforcement learning (RL), shows its effectiveness in handling these challenges. DRL’s ability to operate with minimal labeled training data makes it adaptable for optimizing wireless communication systems. This is especially true for complex and dynamic channel environments. Without total channel model information, DRL constructs wireless channel knowledge by interacting with the environment and receiving rewards. When combined with efficiently designed algorithms, sequentially finding optimal solutions to multi-objective optimization problems becomes a reality when this neural network is combined with efficiently designed algorithms. This approach excels in joint optimization-based networks, such as beamforming design, power allocation strategy, interference coordination, and user distribution model tracking in diverse communication scenarios, showcasing its usefulness in addressing the delicate challenges of RIS-assisted NTNs. With its ability to navigate complex and dynamic channel environments using minimal labeled training data, DRL showcases its superiority over standard schemes. The comparison emphasizes DRL’s efficiency in real-time decision-making for joint optimization tasks, such as transmit beamforming and RIS phase shift matrix optimization. The adaptability and effectiveness of DRL in addressing the intricate challenges of RIS-assisted NTNs stand out from traditional methods, suggesting that it represents a more advanced and efficient approach to channel estimation in this context.

A direct examination of the reciprocity in channel estimation for both uplink and downlink communication with RISs reveals fascinating dimensions rooted in actual scientific understanding [98]. While fully acknowledging the established reciprocity in time division duplexing, introducing RISs as something that breaks the reciprocity introduces genuine intrigue. Research on AI and ML algorithms could be pivotal in mapping channel responses and optimizing RIS behavior based on incident angles and frequency responses, presenting a solid approach to tailoring specific deployment scenarios [98,99]. This fusion of AI, ML, and RIS optimization gives the potential for advancements in wireless communication systems, particularly when considering the actual science behind optimizing phase shifts and channel state information (CSI) in NTN environments [98,99,100].

Integrating AI and ML to realize RISs for communication in HAPs and NTNs presents realistic possibilities based on ongoing research [8]. The article emphasizes the practical application of DL to navigate challenges in modeling the propagation environment. Reducing the dimensions of channel estimation and discerning deployment-specific characteristics can enhance the accuracy of the learning algorithms in local propagation conditions and direct optimal signal reflection on expansive surfaces. This aligns with the complex realities of user locations and the intricate effects of mutual coupling that prove to be cumbersome for traditional modeling techniques [84].

Moreover, integrating AI and ML introduces a functional solution to configure large-scale RIS deployments. This underscores the potential for learning algorithms to bypass the necessity for precise physical modeling, offering efficient solutions in resolving optimization issues related to configuring surfaces with numerous elements [101]. This suggests that AI and ML can streamline the design and operation of RISs, especially in scenarios where conventional mathematical approaches might not be feasible or are time-consuming [101].

The best ML algorithm for optimizing RISs in NTNs, particularly for tasks like optimizing phase shift and CSI, would likely be reinforcement learning, specifically Deep Q-Networks (DQNs). DQNs excel in scenarios where the system needs to learn optimal actions in dynamic and changing environments [102]. In the context of RIS-aided NTNs, where adaptation to real-time conditions is crucial, a DQN’s ability to handle complex decision-making processes and learn from experience makes it a robust choice. The use of AI or ML further gives the capability to navigate the challenges posed by mutual coupling effects and dynamically adjust RIS configurations to align well with the requirements of optimizing phase shift and CSI in an NTN setting.

## 4. Integration with Communication Technologies

### 4.1. Synergy Between RISs and Current, Future Wireless Communication Systems Including 6G

Integrating RISs into existing and forthcoming communication systems, including the anticipated 6G, signifies a transformative leap towards more dynamic, efficient, and robust communication networks [103,104]. The RIS holds immense potential in synergizing with and enhancing current and future communication protocols [105]. This section explores how RISs can be harmoniously integrated with current and future wireless communication systems, mainly focusing on the evolution towards 6G.

1. **Enhancing 5G Networks and Beyond:** As the world gradually transitions from 5G to 6G, RISs are poised to play a crucial role in addressing the limitations of previous generations and enhancing network performance [104]. Fifth-generation networks, characterized by high data rates, low latency, and massive device connectivity, can benefit from RIS’s ability to provide surfaces that improve signal propagation and coverage, especially in urban environments where obstacles are prevalent [103]. RISs can mitigate signal degradation and enhance the efficiency of massive MIMO systems, which are integral to 5G technology [106,107].
2. **Empowering 6G Vision with RISs:** The envisioned 6G networks aspire to provide ubiquitous wireless intelligence, extreme connectivity, and profound integration with AI. RISs inherently align with these goals, offering adaptive environments that can be optimized for various communication needs. By controlling the propagation of electromagnetic waves, RISs can help 6G to achieve its targets of THz communications, extremely low latencies, and seamless integration of terrestrial, aerial, and space networks [103,105].
3. **Synergy with IoT and Machine-Type Communications:** The IoT and Machine-Type Communications (MTCs) are expected to increase under 6G, with billions of devices communicating simultaneously [105]. RISs can enhance these devices’ energy efficiency and reliability by optimizing signal paths and reducing interference, thereby extending the battery life and improving the performance of IoT and MTC networks [108,109]. This is especially crucial in dense networks where traditional methods of ensuring quality of service may fall short.
4. **Facilitating Advanced Beamforming and Spatial Diversity:** Advanced beamforming and exploiting spatial diversity are critical techniques in modern communication systems to improve signal quality and system capacity. RISs can provide a cost-effective means to implement these techniques, offering finer control over the signal propagation environment [110]. By dynamically adjusting the phase shifts of individual elements, RISs can create constructive or destructive interference where needed, significantly enhancing the capabilities of beamforming and MIMO technologies in current and future communication systems [111].
5. **Integrating with Software-Defined Networks (SDNs) and Network Function Virtualization (NFV):** As networks become more software-driven, integrating RISs with SDNs and NFV can lead to highly flexible and adaptable network architectures. RISs can provide physical-layer adaptability, while SDNs and NFV can dynamically adjust network resources and functions [105]. This synergy optimizes real-time network performance, meeting modern communication systems’ diverse and dynamic requirements.
6. **Enabling Energy-Efficient and Sustainable Networks:** As global communication demands surge, so does the energy consumption of communication networks. RISs offer a pathway to more sustainable networks by enhancing signal efficiency and reducing the need for power-intensive transmitters and infrastructure [111]. This aligns with the green communication objectives of 6G, which emphasize sustainability alongside technological advancement.
7. **Security Enhancements:** Security is a paramount concern in modern communication systems. RISs can contribute to more secure communications by selectively enhancing the signal strength in the intended directions while mitigating eavesdropping risks. Moreover, the dynamic nature of RISs can provide an additional layer of security through variable and unpredictable propagation environments, complicating potential interception or jamming attempts [110,112].

The synergy between RISs and current and future communication systems, including the journey towards 6G, is a testament to the ongoing evolution of wireless technology. The RIS is a pivotal technology that enhances signal propagation, system capacity, energy efficiency, and security [112]. It aligns with the ambitious goals of future communication systems, promising to address the burgeoning demand for high-quality, ubiquitous, and sustainable connectivity. As research and development continue to unfold the potentials of RISs, their integration with current and future communication systems is likely to become increasingly central, paving the way for an era of adaptive, controlled wireless communication environments.

### 4.2. RIS’s Potential in Enhancing Radar and Navigation Systems

Including RISs in radar and navigation systems represents a significant technological advancement, promising enhanced performance and new functionalities [110,113,114]. RIS, essentially a surface with the ability to alter EM waves passing through or reflecting off it, can dramatically improve the efficacy and capabilities of radar and navigation systems in several ways.

1. **Signal Enhancement and Path Loss Reduction:** RISs can reflect signals toward specific directions, mitigating path loss and enhancing signal strength at the receiver. RISs can provide alternative non-LOS paths in complex urban environments or terrains where LOS is obstructed, significantly improving coverage and reliability [110].
2. **Target Resolution and Detection:** RISs can contribute to a higher resolution and better target detection in radar systems. By controlling the phase and amplitude of the reflected waves, RISs can help to distinguish between closely spaced objects, enhancing the radar’s ability to detect and track objects with improved accuracy [110,115].
3. **Reducing Interference and Clutter:** Unwanted signals can be mitigated when an RIS is set up correctly. It will reduce interference and improve the SNR [113]. This is particularly helpful in crowded frequency bands such as those used by frequency-modulated continuous-wave (FM-CW) radar or navigation signals, which are prone to interference from multiple sources. By focusing the signal in desired directions and avoiding undesired ones, RISs can significantly reduce clutter and enhance the system’s performance.
4. **Dynamic Beamforming and Steering:** Traditional radar and navigation systems require mechanical steering of antennas or complex phased array systems for beamforming. RISs can provide a more efficient and faster means to control the wavefront, enabling dynamic beamforming and steering without moving parts or expensive arrays [113]. This can lead to systems that are more responsive, precise, and less prone to wear and tear.
5. **Enhanced Multipath Exploitation:** RISs can be strategically leveraged to harness the dynamic multipath propagation environment; a scenario unequivocally deemed detrimental in conventional systems [110,116]. By meticulously controlling the properties of the surfaces within the environment, RISs can transform the multipath effects from a formidable challenge into a potent advantage, utilizing these phenomena to reinforce system robustness and glean unparalleled insights about the communication environment.
6. **Improved Security:** The ability of an RIS to control electromagnetic waves can also be leveraged to improve the security of multi-static radar and localization signals [104,105]. By ensuring that signals are only strong in intended directions and locations, RISs can make it more difficult for adversaries to intercept or jam the signals in an electronic warfare situation, providing a layer of security that is actively adaptable to threats.

Hence, integrating RISs into radar and navigation systems holds substantial potential, from enhancing signal quality and system performance to opening up new capabilities and improving security. As research intensifies and practical implementations materialize in this field, we may be on the brink of a new era in radar and navigation technology, marked by unprecedented adaptability, enhanced efficiency, and improved robustness. The capabilities of RISs in the beyond 5G (B5G) networks is just beginning to be explored, and the coming years will likely see significant advancements and innovative applications of this promising technology.

## 5. Energy Efficiency and Sustainability

Integrating RISs with HAPs introduces a groundbreaking approach that enhances communication capabilities and addresses some critical energy efficiency and sustainability concerns. The demand for wireless communication has surged, driving the quest for improved communication system performance, especially in remote areas. RIS-enhanced HAPs is among the technologies that offer promising energy efficiency and sustainability solutions in wireless networks.

### 5.1. Energy Efficiency in the Context of RIS and HAP Integration

HAPs are used for various purposes, including telecommunications, surveillance, and environmental monitoring. However, the challenge lies in the energy constraints associated with sustaining operations at such altitudes. Traditional communication systems on HAPs often face limitations in terms of energy consumption, impacting their endurance and overall effectiveness. When equipped with RISs, these platforms can significantly improve their performance metrics, particularly regarding energy efficiency. Energy efficiency is critical to RIS-enhanced HAPs, and researchers have devoted considerable efforts to analyzing its impact [72,102]. They have studied the energy efficiency of RIS-enhanced HAPs in various scenarios, such as multiple-input single-output (MISO) and MIMO networks. RIS technology significantly reduces energy consumption by optimizing various parameters such as time allocation, HAP energy beamforming, receiving beamforming, user transmit power allocation, and the RIS energy reflection coefficient [65]. Analyzing the energy efficiency of RIS-enhanced HAPs involves various factors, such as transmit power, channel conditions, data rates, and the energy consumption of the RIS itself, which must be considered [117]. RIS placement, configuration, and optimization techniques also affect energy efficiency [118].

In the context of RIS-enhanced HAPs, the multi-objective optimization approach involves the joint optimization of various parameters. This includes time allocation, HAP energy beamforming, receiving beamforming, user transmit power allocation, and the energy reflection coefficient of the RIS. This comprehensive optimization scheme aims at maximizing energy efficiency while ensuring fair and equitable distribution of resources among users in the network [119]. The optimization framework offers a significant step towards realizing the full potential of RIS-enhanced HAPs in meeting the ever-increasing demands for wireless communication while fostering sustainability and fairness in wireless networks. Using RIS reduces the number of reflections in the channel, resulting in a favorable channel that requires less transmit power for a given data rate [120].

Optimizing the multi-user transmit beamforming power requirement for the active user set, researchers have proposed computationally efficient algorithms to minimize network power consumption and improve energy efficiency [121]. Moreover, introducing RIS-aided networks has enhanced energy efficiency [122]. In RIS-aided networks, the joint optimization of active beamforming and phase shifter design has significantly improved system performance and energy efficiency [123]. To address the energy efficiency of RIS-enhanced HAPs, various algorithms [65] and optimization techniques [124] have been developed. One approach is optimizing resource allocation by formulating a convex optimization problem and proposing low-complexity sub-optimal algorithms. Optimizing the BS’s precoding matrix and the reflecting surface’s reflection coefficient has maximized the weighted minimum rate for all users to ensure network fairness and minimize energy consumption [65].

#### 5.1.1. Energy Efficiency Through Adaptive Beamforming

One of the primary advantages of incorporating RISs into HAPs is the ability to adapt to the propagation environment dynamically. RISs can effectively modify the phase and amplitude of the incoming EM waves to create constructive interference in desired directions [125]. This beamforming capability means that signals can be focused more accurately on specific receivers, significantly reducing the power required to maintain a reliable communication link, thus enhancing the overall energy efficiency of the HAPs [72,116,125].

#### 5.1.2. Extending Coverage with Reduced Power

Traditional HAPs require significant energy to increase their coverage area, often increasing power consumption [72]. With RISs, HAPs can extend their coverage by intelligently reflecting signals toward blind spots or areas beyond their direct line of sight without additional power expenditure. This ability to manipulate the signal path passively means that more areas can be covered with the same or even less energy, improving the energy efficiency ratio [72,125].

#### 5.1.3. Minimizing Losses Through Passive Elements

RIS consists of numerous low-cost, passive elements that do not require power for signal processing or active radiation. This passive nature significantly reduces the system’s energy consumption [72,116]. Unlike active antenna systems, where each element might require its power source for operation, RISs can manipulate the EM waves impinging upon them without any active energy input, leading to substantial energy savings [126].

#### 5.1.4. Reducing Interference and Enhancing Signal Quality

By controlling the propagation of signals, RISs can also minimize interference, a common problem that requires additional energy expenditure in traditional systems to overcome [103,105]. RISs can improve the SNR by directing signals away from unintended receivers and toward the intended targets. This means that signals require less power to achieve the same level of quality and reliability, further enhancing energy efficiency.

#### 5.1.5. Operational Longevity and Maintenance

RIS’s passive nature and lack of moving parts also mean less wear and tear, leading to longer operational life and reduced maintenance needs. This aspect indirectly contributes to energy efficiency by reducing the energy and resources required for manufacturing, deploying, and maintaining the HAPs [72,126].

#### 5.1.6. Enabling Green Communication Networks

Integrating RISs into HAPs aligns with the broader objective of developing sustainable and green communication networks [126]. By reducing the energy requirements of HAPs, RISs contribute to lowering the overall carbon footprint of communication infrastructure. This is particularly pertinent as the demand for data and connectivity continues to grow exponentially, necessitating solutions that can scale without proportionally increasing energy consumption.

Analyzing the energy efficiency of RIS-enhanced HAPs reveals their potential to optimize power consumption and improve overall system performance [127]. The strategic deployment of RIS technology can enhance the coverage and capacity of wireless networks while reducing the energy consumption of BSs [124]. Hence, the energy efficiency of RIS-enhanced HAPs is multifaceted, stemming from the ability to direct and manipulate signals efficiently, extend coverage with minimal energy increase, and reduce interference. The passive nature of RISs contributes significantly to lowering operational energy demands, making it a promising technology for sustainable and efficient high-altitude communication platforms. As technological advancements continue to unlock the potential of RISs, it is expected that HAPs will become even more energy-efficient, paving the way for an era of more sustainable communication and monitoring solutions in the stratosphere.

### 5.2. Sustainable Practices in Deploying RIS Technology

Sustainable practices are crucial in deploying RIS technology in HAPs and are paramount to ensure long-term environmental benefits. These practices include energy-efficient hardware design, optimized power management algorithms, integration of energy-harvesting technologies [67], and the use of renewable energy sources for HAPs, such as solar or wind energy [127]. The deployment of RIS technology also involves considering the environmental impact and resource management [128]. This includes minimizing electromagnetic pollution and reducing the exposure of the end user to electromagnetic fields [129]. Moreover, energy-efficient algorithms and optimization techniques can be applied to maximize the energy harvested by RIS-enhanced HAPs, leading to a more sustainable and eco-friendly operation [130].

Various approaches have been recently explored to ensure the long-term sustainability of RIS-enhanced HAPs. Factors such as energy consumption and resource allocation optimization have been considered [124]. These practices include optimizing power allocation, beamforming strategies, and integrating renewable energy sources into the HAP’s infrastructure. For example, a novel mechanism for a new element on–off to improve the scalability and energy efficiency of RIS-enhanced HAPs is presented in [122]. This mechanism allows RIS elements to selectively activate or deactivate based on channel conditions and user requirements, reducing unnecessary energy consumption and improving overall system sustainability. Furthermore, implementing energy-saving mechanisms, such as sleep mode and dynamic power control, can further enhance energy efficiency in RIS-enhanced HAPs.

Developing mitigation strategies to tackle the environmental impact based on assessment must be performed to identify potential risks. These strategies include considering the materials used in manufacturing RIS elements, ensuring proper disposal and recycling processes, and minimizing the overall carbon footprint of RIS-enhanced HAPs [128].

Deploying RIS technology in HAPs will foster a more eco-friendly and energy-efficient wireless communication system. This can be achieved through several approaches, such as follows:

- **Community pricing:** Rather than individual householders bearing the cost of RIS deployment, community developers can price the deployment of RISs [131]. This approach reduces complexity and promotes collective responsibility in implementing RIS technology.
- **Function pricing:** Customizing the deployment and size of RISs based on different communication needs can optimize resource allocation and promote efficient usage of RIS technology.
- **Renewable energy integration:** Integrating renewable energy sources, such as solar or wind power, into RIS deployment can reduce reliance on traditional energy sources and promote sustainability by reducing greenhouse gas emissions.
- **Lifecycle assessment:** Conducting a thorough lifecycle assessment of RIS technology, including its manufacturing, deployment, maintenance, and disposal, helps to identify areas where environmental impact can be minimized and sustainability practices can be improved.
- **Standardization:** Developing industry-wide standards for RIS technology ensures interoperability and compatibility, reducing unnecessary duplication of resources and promoting efficient deployment practices.
- **Recycling and reuse:** Implementing processes for recycling and reusing materials used in RIS technology, such as electronic components, can reduce waste and promote sustainability.
- **Energy-efficient infrastructure:** Designing RIS systems with energy-efficient components and technologies, such as low-power-consumption RIS types, can reduce the RIS technology’s overall energy consumption and environmental impact.

## 6. ML-Driven RIS-NTN Systems: Future Perspectives and Challenges

The RIS is a groundbreaking technology in wireless communication networks, potentially significantly changing how data are transmitted, received, and handled [132]. Through the intelligent reflection and manipulation of EM waves, RISs can improve signal strength, reduce interference, and enhance network performance across various settings [120,133]. However, conventional optimization strategies relying on CSI encounter intrinsic constraints that limit their effectiveness in fully utilizing the potential of RIS technology. These limitations arise from wireless channels’ intricate and fluctuating nature and the computational burden linked to real-time adjustments and optimization. Therefore, a critical requirement is for more effective and adaptable optimization approaches to leverage the complete capabilities of RIS-enhanced networks [15,134,135].

Recently, ML has become a promising method for overcoming the optimization obstacles that RIS technology encounters. Through utilizing data-driven observations and adaptable algorithms, ML methods can enhance RIS performance in real-time by constantly adjusting phase shifts and beamforming patterns to maximize signal strength and reduce interference [136,137]. Advancements in optimization driven by ML have shown considerable enhancements in RIS performance in various areas, such as mapping, propagation modeling, localization [138], signal processing, and resource allocation. ML algorithms can dynamically optimize RIS setups to accommodate evolving environmental conditions and user needs by analyzing large datasets and identifying intricate wireless channel behavior [126,135].

Nevertheless, while ML-driven optimization holds potential, various challenges and research hurdles must be tackled. One notable difficulty involves the requirement for extensive datasets to train ML models. Moreover, real-time optimization presents significant computational complexity, and ensuring the resilience of ML algorithms in dynamic and uncertain wireless environments is also a significant obstacle [15,126]. Furthermore, integrating ML with RIS technology necessitates addressing interoperability issues and guaranteeing smooth integration with current network architectures.

Looking to the future, the combination of ML and RIS technology has the potential to transform wireless communication networks, leading to more effective, adaptable, and intelligent systems. By tackling the issues associated with conventional optimization methods and leveraging data-driven insights, ML-driven RIS optimization offers groundbreaking improvements in network performance, dependability, and scalability. The convergence of ML and RIS marks a significant milestone in advancing wireless communication networks. Through the utilization of adaptive optimization and intelligent reflection principles, this ML-driven RIS technology offers an opportunity to unlock the complete capabilities of future wireless networks, thereby influencing connectivity in the digital era [16,134,139].

Several future challenges for implementing RISs in NTNs using ML techniques are presented as follows:

**Resource-efficient RL Methods:** Despite promising results, one significant challenge is developing accurate RL methods with moderate computational and energy demands suitable for aerial platforms. The focus should be on ensuring that RL techniques comply with the resources available to the aerial platforms, especially considering real-world scenarios’ dynamic and stochastic nature.

**Integration with Advanced Technologies:** The evolution of NTNs to support super-high-definition (SHD) and extremely high-definition (EHD) videos with super-high throughput demands and ultra-reliable low-latency communications presents a complex challenge. Integrating RISs into this landscape becomes a multifaceted task. ML methods must adapt to efficiently support 3D virtual reality, space and underwater connectivity, autonomous devices, backscatter communication, and energy harvesting, further adding to the intricacy of the problem.

**Discrete Phase Shift Optimization:** The emergence of discrete phase shift models due to hardware limitations poses a significant challenge. Current techniques for solving discrete phase shift problems, such as quantization or brute-force searching, have performance degradation or complexity limitations. The need for advancements in optimization algorithms or adapting DRL algorithms to provide more efficient solutions is pressing, as more than the current solutions are needed to meet the demands of the problem.

While RL shows promise for attaining optimal control strategies in wireless communications, there is a need to create precise RL techniques that are both computationally efficient and energy-efficient, particularly for aerial systems. These methods, once perfected, can accurately simulate real-world multi-agent uncertain situations and seamlessly interface with 3D virtual reality to fulfill requirements for SHD, EHD, high-throughput, and ultra-reliable low-latency communications, offering a bright future for this field. The concurrent optimization of active and passive beamforming poses significant challenges, particularly when dynamically sensing channel state information in NTNs with RIS assistance. New RL architectures such as soft-deep deterministic policy gradient (DDPG) algorithms have been proposed to address these challenges, but further investigation is needed to improve network performance in real-time and adapt to the evolving variations in RIS reflective element amplitudes [140,141].

Developing simplistic methods that can deliver results comparable to exhaustive search techniques continues to present a significant challenge. While traditional optimization strategies such as the greedy algorithm hold potential, it is crucial to enhance the efficiency of RL algorithms in wireless applications in order to address these challenges effectively.

## 7. Conclusions

In this paper, we have summarized the latest research activities on the emerging field of RIS-assisted NTN-based 6G communications. By addressing critical user needs such as enhanced security, energy efficiency, expanded range, and high data rates, the integration of RISs has been demonstrated as a key enabler of future wireless communication systems. The synergy between RISs and MIMO systems, as well as advanced radio communications, highlights the importance of AI and ML in optimizing RIS-based beamforming. These technologies work together to solve pressing scientific and engineering challenges while promoting sustainability in NTN operations. The findings in this paper emphasize the pivotal role RISs will play in unlocking the full potential of NTNs, driving the evolution of next-generation wireless technologies. By shedding light on the benefits of RISs for 6G NTNs and proposing future research directions, this work offers a comprehensive understanding of how RISs can revolutionize the field and catalyze the development of highly efficient and innovative wireless communication systems. 

## Figures and Tables

**Table 1:** Comparative analysis of various technologies with the RIS.

Attribute	RIS	Massive MIMO	Relay	Backscatter
Operating Mechanism	Passive/Active, reflection	Active, transmission/reception	Active, reception and transmission	Passive, reflection
Duplex	Full	Half/full	Half/full	Full
No. of RF Chains	0	N	N	0
Hardware Cost	Low	Very high	High	Very low
Energy Consumption	Low	Very high	High	Very low

## Data Availability

Data are contained within the article.

## Abbreviations

The following abbreviations are used in this manuscript:

Sixth Generation	6G
Artificial Intelligence	AI
Base Station	BS
Deep Deterministic Policy Gradient	DDPG
Deep Learning	DL
Deep Q-Network	DQN
Deep Reinforcement Learning	DRL
Deep Space Network	DSN
Electromagnetic	EM
Extremely High Definition	EHD
Full-Duplex	FD
High-Altitude Platform	HAP
Integrated Satellite	IS
Internet of Things	IoT
Internet of Vehicles	IoV
Line of Sight	LoS
Low Earth Orbit	LEO
Machine Learning	ML
Machine-Type Communication	MTC
Markov Decision Process	MDP
Microelectromechanical System	MEMS
Multiple-Input and Multiple-Output	MIMO
Network Function Virtualization	NFV
Non-Orthogonal Mutual Authentication	NOMA
Non-Terrestrial Network	NTN
Orthogonal Frequency-Division Multiplexing	OFDM
Positive Intrinsic Negative	PIN
Radio Frequency	RF
Reconfigurable Intelligent Surface	RIS
Reinforcement Learning	RL
Signal-to-Noise Ratio	SNR
Software-Defined Network	SDN
Super High Definition	SHD
Terahertz	THz
Terrestrial RIS	TRIS
Terrestrial Network	TN
Unmanned Aerial Vehicle	UAV
